# Clinical Outcomes of COVID-19 Patients with Pre-existing, Compromised Immune Systems: A Review of Case Reports

**DOI:** 10.7150/ijms.50537

**Published:** 2020-10-18

**Authors:** Tanner Corse, Linda Dayan, Sydney Kersten, Fortunato Battaglia, Stanley R Terlecky, Zhiyong Han

**Affiliations:** 1 Hackensack Meridian School of Medicine, Nutley, NJ 07110, USA; 2 Department of Medical Sciences, Hackensack Meridian School of Medicine, Nutley, NJ 07110, USA.

## Abstract

In the ongoing COVID-19 pandemic, all COVID-19 patients are naïve patients as it is the first-time humans have been exposed to the SARS-CoV-2 virus. As with exposure to many viruses, individuals with pre-existing, compromised immune systems may be at increased risk of developing severe symptoms and/or dying because of (SARS-CoV-2) infection. To learn more about such individuals, we conducted a search and review of published reports on the clinical characteristics and outcomes of COVID-19 patients with pre-existing, compromised immune systems. Here we present our review of patients who possess pre-existing primary antibody deficiency (PAD) and those who are organ transplant recipients on maintenance immunosuppressants. Our review indicates different clinical outcomes for the patients with pre-existing PAD, depending on the underlying causes. For organ transplant recipients, drug-induced immune suppression alone does not appear to enhance COVID-19 mortality risk - rather, advanced age, comorbidities, and the development of secondary complications appears required.

## Introduction

The ongoing COVID-19 pandemic is caused by the zoonotic coronavirus SARS-CoV-2 1, which primarily infects cells in the respiratory system, including nasal ciliated epithelial cells and goblet/secretory cells, unciliated epithelial cells in the respiratory tracts, type II alveolar pneumocytes, and endothelial cells of the microvasculature of lungs 2, 3. These cells express the angiotensin converting enzyme 2 (ACE2) as a transmembrane protein in the plasma membrane, and SARS-CoV-2 binds to the extracellular domain of ACE2 to gain entrance into the cell to initiate an infection 3. Some SARS-CoV-2-infected individuals are asymptomatic 4, but most infected persons initially develop upper respiratory infection symptoms, such as fever, cough, sore throat, fatigue, and myalgia, with later development of viral pneumonia with accompanying dyspnea 5, 6. Most symptomatic COVID-19 patients recover with supportive care in hospitals; however, a small but significant percentage of COVID-19 patients are at risk of developing acute respiratory distress syndrome (ARDS) or respiratory failure 7, 8. Those particularly at risk are elderly COVID-19 patients (>60 years) and those with underlying comorbidities, such as diabetes, hypertension, and chronic obstructive pulmonary disease 7, 8. Such patients often require intensive care unit (ICU) care and oxygen therapy including mechanical ventilation 7, 8. These are critically ill COVID-19 patients and the mortality rate among them is high.

An analysis of the clinical outcomes of 44,672 COVID-19 patients in China suggested an overall mortality rate of 2.3%, but it was 8.0% among those aged 70-79 years, and 14.8% among those aged >80 years 9. In Italy, the study by Onder et al found that the overall mortality rate is 7.2%, with a mortality rate of 0% for those aged <29 years, 0.3% for those aged 30-39 years, 0.4% for those aged 40-49 years, 1% for those aged 50-59 years, 3.5% for those aged 60-69 years, 12.8% for those aged 70-79 years, and 20.2% for those aged >80 years 10. Analysis by the United States Center for Disease Control and Prevention (CDC) of the clinical outcomes of COVID-19 patients in the United States between February 12 and March 16, 2020 showed that the mortality rate of COVID-19 patients also varies with age: <1% among patients aged 20-54 years; 2% among patients aged 55-64 years; 7% among patients aged 65-84 years; and 18.5% among patients ≥85 years 11 These analyses indicate that the risks of developing severe COVID-19 and dying from it are higher in persons of advancing age. Postmortem studies of deceased COVID-19 patients suggest that death from COVID-19 results primarily from respiratory failure due to extensive diffuse alveolar damage, and pulmonary microvascular thrombosis 12-15.

Thus far, most evidence suggests that an overly active immune system in COVID-19 patients causes activation of a disproportionately high number of pathogenic T cells and inflammatory monocytes/macrophages, resulting in the development of an inflammatory cytokine storm. This causes severe damage of alveolar epithelial cells and endothelial cells of microvasculature in the lungs, culminating in ARDS and respiratory failure 5, 16, 17. Therefore, treatment of COVID-19 needs to achieve activation of anti-SARS-CoV-2 immunity, and at the same time prevention of inflammatory damage and thromboembolism in the lungs.

One of the many unanswered questions regarding COVID-19 is how the naïve immune system reacts, adapts, and mounts an effective defense against the SARS-CoV-2 virus. In comparison to COVID-19 patients with mild to moderate symptoms, those with severe symptoms had significantly decreased counts of CD3^+^, CD4^+^, and CD8^+^ T cells 8, 18-20, decreased numbers of B cells and natural killer (NK) cells 20, and exhaustion of functional T cells 19, 21. Additionally, there is evidence of decreased numbers of CD45^+^ T cells and CD19^+^ B cells 18. These findings collectively suggest that the development of severe COVID-19 is likely due to weakening of both T-cell-mediated and B-cell-mediated immunities. Moreover, these findings raise concerns about what would happen to immunocompromised individuals if they are infected by the SARS-CoV-2 virus. Would they be predisposed to developing severe COVID-19, and thus manifest a higher mortality rate than others? From this perspective, it is especially challenging for physicians to develop treatment strategies for SARS-CoV-2-infected patients on maintenance immunosuppressants, as it may require making a difficult decision of continuing or discontinuing the drugs. Continuation of immunosuppressants may very well impair the development of immunity against SARS-CoV-2, whereas their discontinuation may exacerbate non-COVID-19 symptoms and permit development of a pro-inflammatory cytokine storm. To evaluate differences in the clinical characteristics and outcomes of COVID-19 patients with a pre-existing compromised immune system, we conducted a search and review of relevant published reports.

## Methods

**Figure 1:**
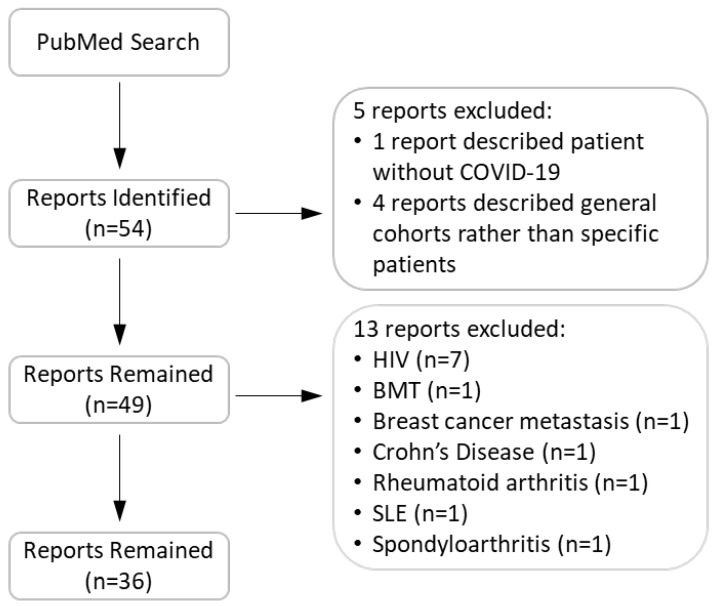
Flow chart of search, inclusion, and exclusion of articles.

We conducted a search in the PubMed® database for reports that describe COVID-19 patients with pre-existing compromised immune systems. Our search key words contained COVID-19 in combination with one of the following: immunocompromised, rheumatoid, arthritis, lupus, Crohn's, celiac, HIV, AIDS, lung transplant, heart transplant, liver transplant, kidney transplant, renal transplant, agammaglobulinemia, immune deficiency, and immune suppressant. Figure 1 shows our search flow. In total, we identified 53 relevant case reports. We subsequently excluded 18 reports as they were opinion articles or contained insufficient information to indicate compromised immunity or lacked a history of immunosuppressant use. The remaining articles included 126 patients. We reviewed the clinical characteristics of these patients and their outcomes. Clinical outcomes included: i) a patient either was treated as an outpatient and recovered, or was treated in the hospital and ultimately discharged (positive outcomes); ii) the patient remained hospitalized at the time of the case's publication; or iii) death ensued prior to publication of the case. The information concerning individual patients in the reports we reviewed is described in Tables 1, 2, and Supplementary Table 1. During the review, we noticed that detailed laboratory values were not reported for most patients. Therefore, the Tables do not contain such information.

## Results

### Clinical Outcomes of Patients with Primary Antibody Deficiency and COVID-19

There were 3 reports 22-24 describing a total of 10 COVID-19 patients with pre-existing PAD (Table 1). Of these, 6 patients had common variable immunodeficiency (CVID), 3 had X-linked agammaglobulinemia (XLA) due to loss-of-function mutations in the Bruton tyrosine kinase (BTK), and 1 patient had autosomal recessive agammaglobulinemia (ARA), the etiology of which is unknown. The ages of these patients ranged from 26 to 59 years. Three patients were female and 7 were male. All patients had been on maintenance immunoglobulin therapy to prevent common infections. For their SARS-CoV-2 infection, 9 patients received treatment with hydroxychloroquine, and 7 of these 9 patients received concomitant antivirals (most commonly lopinavir plus ritonavir) with or without antibiotics (Table 1).

Of the 6 patients with pre-existing CVID, 3 (50%) patients achieved a positive outcome, 2 patients had their conditions improved, and 1 patient died (Table 1). The patient who died was a 59-year-old female. She was one of the 4 patients who developed severe respiratory symptoms that required ICU care and ventilation, and she received treatment with tocilizumab before death (Table 1). The COVID-19 patient with pre-existing ARA was treated as an outpatient and achieved a positive outcome. Of the 3 patients with pre-existing XLA, 2 patients achieved a positive outcome after a short hospital stay, and 1 remained hospitalized with an improved condition at the time of the case publication (Table 1). Overall, there was no clear link between a specific comorbidity (or a group of comorbidities) and the clinical outcomes for the 10 patients (Table 1).

### Clinical Outcomes of Heart or Liver, or Lung Transplant Recipients with COVID-19

Ten reports 25-34 described a total of 10 heart transplant recipients with COVID-19 (9 were male), 11 liver transplant recipients with COVID-19 (8 were male), and 2 lung transplant recipients with COVID-19 (Table 2). On admission, most patients presented with common symptoms, such as cough, fever, and dyspnea. Patient ages ranged from 38 to 77 years, and the average time since the transplant was 11.97 years. Sixteen (70%) patients had comorbidities, with hypertension and diabetes being the most prevalent. All patients were on maintenance immunosuppressant therapy before hospitalization, with tacrolimus and MMF most often employed, followed in use by glucocorticoids, and cyclosporine (Table 2). Of the 23 patients, 17 (73.9%) continued to use immunosuppressants during hospitalization, while 6 (26.1%) had their immunosuppressants discontinued. None of these reports indicated any signs of organ rejection in patients during hospitalization. Most patients received treatment with antivirals and hydroxychloroquine during hospitalization, and some received additional drugs including antibiotics, interferon, and tocilizumab (Table 2).

The ages of the heart transplant recipients with COVID-19 ranged from 38 to 77 years, and the average time since the transplant procedure was 13 years. One patient was treated as an outpatient with a positive outcome. Nine (90%) patients had at least one comorbidity with hypertension and diabetes being the most prevalent, and 8 of them continued to use immunosuppressants during hospitalization (one patient had immunosuppressants discontinued during the first 11 days and then was back on the drugs for 22 days). During hospitalization, 3 patients developed ARDS. Of these 3, one patient died, another developed additional renal failure, required both ICU care and ventilation, and ultimately died, and the third achieved a positive outcome (Table 2). Overall, 8 (80%) patients achieved a positive outcome, and thus the overall mortality rate was 20%. Of special note was that one patient who was a recipient of both heart and kidney transplants; although he required ICU care, he achieved a positive outcome after a 10-day hospitalization (Table 2). However, there was no clear connection between the presence of a specific comorbidity (or a group of comorbidities) and the clinical outcomes (Table 2). The high rate of positive outcomes suggests that heart transplant recipients with COVID-19 on immunosuppressants are not at an increased risk of mortality unless the patient develops complications such as ARDS and/or requires ICU care and ventilation.

The ages of the 11 liver transplant patients ranged from 46 to 79 years, and the average time since transplantation surgery was 11.53 years (Table 2). Of the 11 patients, 6 (55%) had at least one comorbidity, with diabetes and hypertension being the most prevalent, and 9 (82%) continued to use immunosuppressants during hospitalization. Two patients were treated as outpatients (1 patient received hydroxychloroquine), and both achieved a positive outcome (Table 2). Among those hospitalized, 3 patients developed ARDS and 1 patient developed pneumonia and cholestasis (Table 2). Overall, 7 (64%) patients (including the two outpatients) achieved a positive outcome, 1 patient remained hospitalized at the time of the case publication (this patient developed ARDS and required both ICU care and ventilation), and 3 patients died. Of the 3 patients that died, 1 aged 72 years developed ARDS, 1 aged 73 years developed ARDS and required ICU care and ventilation, and 1 aged 59 years developed pneumonia, cholestasis, and required ICU care and ventilation. Thus, the overall mortality rate was 27.3%. It should be noted that old age by itself did not appear to be a risk of mortality because two patients aged 79 years and 76 years did not develop secondary complication and achieved a positive outcome (Table 2), and additionally, there was not a clear connection between the presence of a specific comorbidity (or a set of comorbidities) and the clinical outcomes (Table 2). Therefore, liver transplant recipients sustained on immunosuppressants do not seem to be at an increased risk of mortality from COVID-19, but rather the subsequent development of complications such as ARDS and the requirement for ICU care and ventilation place the patient at high risk of mortality.

As shown in Table 2, there were 2 female lung transplant recipients with COVID-19. Both patients were in their 50s and developed relatively mild symptoms. Both had been on glucocorticoids plus other immunosuppressants prior to infection. One patient was treated as an outpatient with a mild COVID-19 disease course resulting in a positive outcome. She continued to use immunosuppressants and did not receive other drugs. The other patient was hospitalized with a mild COVID-19 course, during which she continued to use immunosuppressants and received treatment with antibiotics and a bronchodilator. This patient achieved a positive clinical outcome as well (Table 2).

### Clinical Outcomes of Kidney Transplant Recipients with COVID-19

In total, there were 21 reports 35-55 describing a total of 93 kidney transplant recipients with COVID-19 (Supplementary Table 1). Patient ages ranged from 21 to 80 years, the average time since transplant procedure was 8.3 years, 75% were male, and 25% were female. On admission, most patients presented with fever and cough. A total of 66 (71%) patients had at least one comorbidity, with hypertension (82%) and diabetes (34%) being the most common. None of these reports indicated any signs of kidney rejection in patients during hospitalization. Of the 93 patients, the duration of hospitalization for 36 patients were reported (Supplementary Table 1), and most of these individuals achieved a positive outcome.

As shown in Supplementary Table 1, all patients were on maintenance immunosuppressants before hospitalization for SARS-CoV-2 infection: 88% on a calcineurin inhibitors (CNI), 77% on MMF, and 76% on a glucocorticoid. Tacrolimus was the most used CNI, prednisone was the most used glucocorticoid, and the triple therapy of tacrolimus-MMF-prednisone was the most common combinatorial maintenance therapy. Other immunosuppressants used include cyclosporine, mTOR inhibitors, azathioprine, belatacept, adalimumab, and leflunomide.

Also shown in Supplementary Table 1, 89 of the 93 kidney transplant recipients with COVID-19 continued to use some type of immunosuppressant during hospitalization. Of the 89 patients, 80 (86%) patients used glucocorticoids during their hospitalization, with 67 (85%) of them having received glucocorticoids prior to admission as well. During hospitalization, many patients received treatments with antivirals (mainly lopinavir and ritonavir), hydroxychloroquine, and antibiotics.

During hospitalization, 5 (5.6%) patients acquired nosocomial infection (1 patient tested positive for *pseudomonas*, 1 patient was positive for *Enterococcus* in the urine, 2 patients were positive for *Klebsiella pneumoniae* in the urine, and 1 patient was positive for *Eschericia coli* in the urine). Two (2.2%) patients developed ARDS, 7 (7.9%) patients developed acute kidney injury (AKI), 11 (12%) patients required ICU care during hospitalization, and 15 (16%) patients required ventilation.

The 89 kidney transplant recipients with COVID-19 who continued to use immunosuppressants made additional analysis possible. Therefore, we have extracted the information from Supplementary Table 1 and summarized it in Table 3 to determine if there is a relationship between clinical outcomes as measured against age, gender, comorbidity, secondary complications, and requirement for ICU care and/or ventilation.

As shown in Table 3, of the 89 patients, 43 (48%) achieved a positive clinical outcome, 32 (35.9%) remained hospitalized at the time of their case publication, and 15 (16.9%) died. However, the mortality rate among those without comorbidities was 7.4%, while it was 20% among those with at least one comorbidity (Table 3). Thus, it indicates a substantial comorbidity-associated increase in mortality risk. The ratio of male patients to female patients was 3.04, and the mortality rate for female patients was 22.7%, and 14.9% for male patients. When the 89 patients are divided by age groups, the clinical outcomes reveal a higher mortality rate for older age groups compared to younger age groups: in the ≤39-year age group, the rate of a positive outcome was 85%, the rate of remaining in hospital was 15%, and the death rate was 0%; in the 40-49 year age group, the rate of a positive outcome was 41.7%, the rate of remaining in hospital was 58.3%, and the death rate was 0%; in the 50-59-year age group, the rate of a positive outcome was 45.8%, the rate of remaining in hospital was 33.3%, and the death rate was 20.8%; in the 60-69-year age group, the rate of a positive outcome was 25%, the rate of remaining in hospital was 62.5%, and the death rate was 12.5%; in the ≥70-year age group, the rate of a positive outcome was 29.4%, the rate of remaining in hospital was 23.5%, and the death rate was 47.1%. The ultimate outcome rates of patients aged >40 years remain unknown at present time, however, as high numbers of this population were hospitalized at the time of their case publication. Nevertheless, these values indicate that older patents (>50 years) were at high risk of mortality, with the death rate especially high for patients aged >70 years.

In Table 4, we summarize the information concerning the types of comorbidities in relationship to patient ages and clinical outcomes. Consistent with what we see for the heart or liver transplant recipients with COVID-19 (Table 2), there was no strict correlation between a specific comorbidity and clinical outcomes of the kidney transplant recipients with COVID-19 across the age groups.

In Table 5, we specifically summarize the information concerning the comorbidities, secondary complications, and requirement for ICU care and ventilation of those kidney transplant recipients with COVID-19 who died. The data further illustrates that death was not associated with a specific comorbidity (or a specific group of comorbidities), nor a secondary complication or the requirement for ICU care and ventilation.

In summary, kidney transplant recipients with COVID-19 aged ≤ 39 years had the highest rate of positive outcomes at 85%. The mortality rates varied among different age groups: 0% for the ≤49-year age group; 20.8% for the 50-59-year age group, 12.5% for the 60-69-year age group, and 47.1% for the ≥70-year age group. The presence of comorbidities and secondary complications increased the risk of mortality, but death was not associated with a specific comorbidity and/or a secondary complication. It should be noted that the true rates of positive outcome for those patients aged >40 years remain unclear because of the high number of hospitalized patents at the time of their case publication. Therefore, the ultimate outcomes of these patients will alter the mortality rates of patients in the 40-49-year, 50-59-year, 60-69-year, and ≥70-year age groups.

## Discussion

Although the reported patient number is very small, it is nevertheless interesting to note that 4 out of the 6 patients with pre-existing CVID developed severe COVID-19 that required both ICU care and ventilation, and 1 of them died (Table 1); whereas the patients with pre-existing XLA and ARA had non-life threatening COVID-19 (Table 1). Therefore, individuals with CVID may be predisposed to developing a severe COVID-19 course if they are infected with SARS-CoV-2. From this perspective, it is important to note that the CVID is associated with abnormally developed B cells with impaired antibody-producing function 56, whereas XLA and ARA are associated with an extremely low number of, or no, B cells in the circulation due to blockage of the proliferation and development of pre-B cells in the bone marrow 57, 58. As elevation of serum levels of proinflammatory cytokines are believed to drive the development of COVID-19 complications, such as ARDS and respiratory failure 59-62, it suggests that the abnormally developed B cells in the SARS-CoV-2-infected CVID patients may play a pathogenic inflammatory role in the course of the development of the severe respiratory conditions. However, future investigation is required to determine if and what activities of the abnormally developed B cells contribute to the development of severe COVID-19 respiratory conditions.

The recovery of SARS-CoV-2-infected patients with XLA and ARA as well as the SARS-CoV-2-infected patients with CVID indicates the presence of a cellular immunity against SARS-CoV-2. This agrees with findings by others. For example, Wu et al have shown that a small but significant number of patients recovered from COVID-19 lack SARS-CoV-2-neutralizing antibodies 63, and that Liu et al have found similar mortality risks between those COVID-19 patients who tested positive for IgG antibodies against SARS-CoV-2 and those who lacked antibodies against SARS-CoV-2 64. Therefore, those recovered patients with XLA, ARA, or CVID must have an activated T-cell immunity against SARS-CoV-2 virus. In this context, it is relevant to note the presence of SARS-CoV-2-reactive CD4+ T cells in recovered COVID-19 patients 65, 66, and that the development of SARS-CoV-2 reactive CD38+/HLA-DR+/CD4+ T cells strongly correlates with a positive clinical outcome 66. Future studies of the T cell immunity of the patients with XLA or ARA or CVID who recovered from COVID-19 will be needed to identify SARS-CoV-2-reactive T cells that play a critical role in the recovery of these patients from COVID-19.

Recently, Myets et al., published the clinical outcomes of 94 SARS-CoV-2-infected individuals with inborn errors of immunity (IEI), including 53 individuals with primary antibody deficiency, the causes of which include XLA, ARA, and CVID, and some patients with T cell deficiency due to loss-of-function mutations in ZAP70, PGM3, STAT3, and ARPC1B 67. In the study, 10 patients (10.6%) were asymptomatic, 24 patients (25.5%) developed mild symptoms and were treated as outpatients, and 59 patients (62.8%) required hospitalization. Of the hospitalized patients, 29 (49.1%) developed respiratory insufficiency and 13 (22%) required ICU care and invasive ventilation. The overall mortality rate is 9.5% (9/94). The authors concluded that “the risk factors predisposing to severe disease and mortality amongst IEI patients were comparable to the general population.” And they suggested that “certain components of adaptive immunity do not appear to be essential for controlling SARS-CoV2 infection. Rather, these adaptive immune deficiencies may even contribute to a milder course by reducing the immune-mediated sequelae.” Strikingly, 4 of the 9 deceased patients had primary antibody deficiency due to CVID 67, suggesting that a pathogenic inflammatory role for CVID in the development of the severe respiratory conditions. However, it should be noted that these 4 CVID patients were generally older than the rest of the cohort and had pre-existing health conditions, suggesting that they were likely predisposed to severe COVID-19 67.

Our analysis indicates an overall high mortality rate among SARS-CoV-2-infected organ transplant recipients who were continuously on immunosuppressants. However, given the small number of COVID-19 patients with a heart, a liver, or a lung transplant, the statistical significance of the mortality rates for these patients cannot be assessed here. For the kidney transplant recipients with COVID-19 who remained on immunosuppressants, the mortality rate among those ≤49 years was 0% (Table 3), and this outcome occurred despite some patients having at least one comorbidity, such as hypertension and diabetes, that have a known association with high mortality risk for COVID-19 patients in general. However, death occurred among the SARS-CoV-2-infected older (≥50 years) kidney transplant recipients who were on immunosuppressants (Table 3), and it contributed to an overall mortality rate of 16.9% for the SARS-CoV-2-infected kidney transplant recipients on immunosuppressants (Table 3). However, the mortality rate among those without comorbidities was 7.4%, while it was 20% among those with at least one comorbidity (Table 3), indicating a substantial comorbidity-associated increase in mortality risk. Interestingly, our review does not indicate a specific comorbidity (or a group of comorbidities) that is responsible for the increased mortality risk (Tables 4 and 5). Therefore, the comorbidity-associated mortality risk is most likely dependent on additional factors that vary from patient to patient. Nevertheless, a study by Richardson et al., 68 and another study by Cummings et al., 69 of hospitalized COVID-19 patients in the New York City area found an overall mortality rate of 21% and 39%, respectively, and moreover these mortality rates are mostly contributed by the death of old patients with comorbidities. Since the overall 16.9% mortality rate of the SARS-CoV-2-infected kidney transplant recipient on immunosuppressants is attributed to death of older (≥50 years) patients with comorbidities and/or secondary complications (Table 3), the 16.9% mortality rate does not seem to be abnormally high because it is in line with the rates reported by others for different COVID-19 patients populations. Nevertheless, the positive outcome of patients aged ≤49 years as well as some older patients suggests that having an immunosuppressant-compromised immune system alone is not a significant risk factor of mortality, but rather the presence or comorbidities, development of secondary complications are more consequential mortality risk factors.

One unfortunate finding in our review is the exceptionally high mortality rate (47.1%) for SARS-CoV-2-infected kidney transplant recipients aged >70 years (Table 3). Why this is the case is not clear. However, it should be noted that within this group of patients, the clinical outcomes could not be predicted even after accounting for the presence of comorbidities and secondary complications. For example, a 76-year-old kidney transplant recipient with COVID-19, who had the comorbidities of hypertension and IgA nephropathy, who continued to use rapamycin and prednisone, and who received treatment with hydroxychloroquine - still achieved a positive clinical outcome after a mere 10-day hospitalization 26. In contrast, a 70-year-old kidney transplant recipient with COVID-19, who had a single comorbidity (hypertension), and who continued to use prednisone, and received treatment with hydroxychloroquine, lopinavir, and ritonavir, died after an unspecified period of hospitalization 51. Therefore, in the absence of additional information, it is possible to hypothesize that the immune system of older patients, as well as some of the younger patients who died, was overly compromised by the immunosuppressants and could thus no longer effectively defend against SARS-CoV-2 infection. It would be interesting to know from blood samples of these deceased patients (if the samples are available), whether there is any link between immune system marker indices and likelihood of death.

Although, the renal function of a significant number of the SARS-CoV-2-infected recipients with kidney transplantation and on immunosuppressants was not reported, some report did include the baseline renal function as measured by eGFR (estimated glomerular filtration rate) or serum levels of creatinine at the time of admission. Although renal dysfunction as reflected by decreased eGFR or increased serum levels of creatinine was not considered in the published reports as a co-morbidity, it is important to recognize that renal dysfunction of kidney transplant recipients with COVID-19 ought to be recognized as such. Nevertheless, numerous reports that we reviewed showed that patients with a decreased eGFR plus other comorbidities 39, 41, 43, 44, 47, 48, 52, 55 or increased serum levels of creatinine plus other comorbidities 36, 38, 40, 42, 46, 53, 54 still achieved a positive outcome. Interestingly, Sharma et al recently completed a retrospective study in which they compared the clinical outcomes of COVID-19 between solid organ transplant recipients and matched nontransplant patients in their medical center, and they found the need for renal replacement therapy in the organ recipients did not affect the incidence of severe COVID-19 and the rate of short-term death 70. The findings by Sharma et al., further suggest that renal dysfunction in SARS-CoV-2-infected organ recipients does not correlate with clinical outcomes.

In a report 71 of the clinical outcomes of 36 consecutive adult kidney-transplant recipients who were infected by SARS-CoV-2, Akalin and colleagues found an overall mortality rate of 28% at 3 weeks, but a mortality rate of 64% among 11 patients who were intubated, indicating a respiratory complication-associated significant increase in mortality rate 71. However, this report neither provide the outcome of individual patient nor specific information on individual patient. In another report 72, Katz-Greenberg et al., described the clinical outcomes of 20 kidney-transplant recipients (ages 30 to 73 years) who were infected by SARS-CoV-2, and showed that only 3 patients (2 males aged 72 and 73 and 1 female aged 63) died, suggesting a 15% mortality that is related to advancing age 72, which agrees with our review of the published case reports.

Nevertheless, it seems intuitive for organ transplant recipients to discontinue immunosuppressants after they become infected with SARS-CoV-2 virus - presumably to allow the body to increase the immune activities to fight the infection. Our review of the positive clinical outcomes of a significant number of organ transplant recipients with COVID-19 suggests that a weaker immune system may not necessarily be a risk factor of mortality. This point is supported by the recent findings by Long et al., showing that in comparison to age- and gender-matched symptomatic COVID-19 patients of similar viral loading, the SARS-CoV-2-infected asymptomatic individuals have a significantly weaker immune response as evidenced by lower IgG levels, lower levels of SARS-CoV-2 neutralization antibodies, and reduced levels of pro-inflammatory cytokines in serum 73. The findings by Long et al., suggest that a weaker immune response does not necessarily result in development of COVID-19 symptoms in SARS-CoV-2-infected persons, but rather it seems necessary to prevent over activation of immune cells leading to the development of a pro-inflammatory cytokine storm, which is known to cause severe lung damage and promote life-threatening respiratory failure in COVID-19 patients. Therefore, it is possible that one reason why SARS-CoV-2-infected young kidney transplant recipients, and some SARS-CoV-2-infected heart and liver transplant recipients, achieved positive outcomes is that their immune systems happened to be compromised by the immunosuppressants to an extent that a proinflammatory storm could not manifest itself, but the residual immunity was sufficient to fight the virus. Perhaps again, a retrospective analysis of blood samples of these organ recipients with COVID-19 would shed light on the relationship between an immunosuppressant-compromised immune system and clinical outcomes. Alternatively, COVID-19 animal models 74-76 may be used to assess how the use of immunosuppressants affects the clinical outcomes of COVID-19.

Our review of published reports has enhanced our understanding of the outcomes of COVID-19 patients with pre-existing, compromised immune systems. However, it has raised numerous issues that warrant future investigation. Specifically, how it is that a compromised immune system, be it a compromised humoral immunity or immunosuppressant-compromised immune system, does not seem to prevent a positive outcome. The findings do suggest that for SARS-CoV-2-infected organ transplant recipients on maintenance immunosuppressants, the decision of whether they should continue to use immunosuppressants should be individualized. As SARS-CoV-2-infected young kidney transplant recipients who continued to take immunosuppressants during hospitalization fared relatively well (Table 3) - it would be reasonable to suggest that those currently hospitalized may continue to use immunosuppressants at the regular doses or reduced dose. (Of course, these are broad generalizations subject to review by physicians overseeing individual patients.) For older patients, it should be determined on a case-by-case basis, perhaps through a gradual dose-reducing process, decreasing the immunosuppressants to a range that does not worsen COVID-19 symptoms and also does not allow high immune reactivity against the transplanted organs. Of course, the laboratory values for all patients should be closely monitored for better understanding of the relationship between a compromised immune system and the outcomes of SARS-CoV-2 infections. Though the information outlined here provides provisional evidence of the effect of immunosuppression on COVID-19 clinical outcomes, more information is needed on this subject moving forward to better understand of the mechanisms of SARS-CoV-2 infection and how COVID-19 can best be treated in humans.

## Supplementary Material

Supplementary table.

## Figures and Tables

**Table 1:** Characteristics and clinical outcomes of patients with hypoglobulinemia who were infected with SARS-CoV-2

Patient Age and Gender	Disease	Immunoglobulin Level* or B-cell Count	Ig Therapy	Comorbidity	Symptoms on Admission	In-Hospital Drugs	Use of ICU/Vent	Length of Hospital Stay (days)	Clinical Outcome	Ref
53 (F)	CVID	IgG (1710 mg/dL) IgA (<7 mg/dL) IgM (33 mg/dL)	Yes	Bronchiecstasis Breast cancer Hypothyroidism Sjogren's disease	Chest pain, chills, dyspnea, fatigue, fever, myalgias,	HCQ Ceftriaxone Doxycycline	Y/Y	Not recorded	Discharged	22
59 (F)	CVID	IgG (897 mg/dL) IgA (30 mg/dL) IgM (33 mg/dL)	Yes	Chronic sinusitis Chronic bronchitis Chronic gastritis	Dyspnea, fever	Tocilizumab	Y/Y	20	Death	23
32 (F)	CVID	IgG (500 mg/dL) IgA (0 mg/dL) IgM (153 mg/dL)	No	Endometriosis Celiac-like disease Allergy Melanoma	Cough, dyspnea, fever	HCQ, Darunavir Ritonavir Tocilizumab	N/Not recorded	16	Condition improved	23
57 (M)	CVID	IgG (550 mg/dL) IgA (40 mg/dL) IgM (44 mg/dL)	Yes	HTN Asthma Obesity	Cough, dyspnea, fever, myalgias	HCQ Lopinavir Ritonavir Remdesivir Glucocorticoid Vancomycin Meropenem Linezolid Caspofungin Cotrimoxazole	Y/Y	25	Discharged	23
52 (M)	CVID	IgG (662 mg/dL) IgA (11 mg/dL) IgM (8 mg/dL)	Yes	Immune thrombocytopenia, Polyclonal lymphoproliferation Recurrent infections Interstitial lung disease	Dyspnea, fever	HCQ Lopinavir Ritonavir Azithromycin	N/N	21	Discharged	23
41 (M)	CVID	IgG (700 mg/dL) IgA (10 mg/dL) IgM (30 mg/dL)	Yes	Recurrent infections, Pneumonia Recurrent sinusitis	Cough, dyspnea, fever	HCQ Lopinavir Ritonavir Piperacillin Tazobactam Tocilizumab Remdesivir	Y/Y	19	Condition improved	23
53 (M)	ARA	IgG (750 mg/dL) IgA (0 mg/dL) IgM (0 mg/dL)	Yes	COPD Bronchiecstasis	None	HCQ Darunavir Cobicistat Antibiotics	N/N	0	Outpatient	23
34 (M)	XLA	IgG (800 mg/dL) IgA (0 mg/dL) IgM (0 mg/dL)	Yes	Bronchiecstasis Skin infection Respiratory infection	Fever	HCQ Lopinavir Ritonavir Antibiotics	N/N	3	Discharged	23
34 (M)	XLA	B cell (absent)	Yes	Perianal abscess	Cough, fever	HCQ Ig Lopinavir Ritonavir	N/N	14	Discharged	24
26 (M)	XLA	B cell (absent)	Yes	None	Anorexia, cough, fatigue, vomiting	HCQ Ig Azithromycin Ceftriaxone	N/N	Not provided	Condition improved	24

*The reference intervals for healthy adults: IgG 700-1600 mg/dL, IgA 70-400 mg/dL, and IgM 40-230 mg/dL [Dati, https://pubmed.ncbi.nlm.nih.gov/8831057/] Ig: immunoglobulins; COPD: Chronic obstructive pulmonary disease; HCQ: hydroxychloroquine; HTN: hypertension; ICU: Intensive care unit; XLA: X-linked agammaglobulinemia; ARA: autosome recessive agammaglobulinemia.

**Table 2:** Characterization and clinical outcomes of heart, liver, and lung transplant recipients with COVID-19

Patient Age and Gender)	Organ (years)	ISx History	Comorbidity	Symptoms on Admission	ISx Used in Hospital	Other Medicines used in Hospital	In-Hospital Complications	ICU/Vent	Length of Hospital Stay (days)	Clinical Outcome	Ref.
59 (F)	Heart(8)	TAC MMF	HPN DM CKD	Cough, fever, dyspnea	TAC (removed on day 6) MMF	HCQ, tocilizumab, IVIG, lopinavir, ritonavir, antibiotics	ARDS, Renal failure	Y/Y	10	Death	25
63 (M)	Heart(17.9)	Pred Cysp MMF	HPN DM CVD CKD Cancer	Cough, fever, dyspnea	Pred MMF	HCQ, lopinavir, ritonavir, interferon-beta	ARDS	*/N	10	Death	26
75 (M)	Heart(20)	Cysp MMF	HPN DM CKD CVD	Cough, fever	Cysp	HCQ, tocilizumab, methylprednisolone		N/N	8	Discharged	25
51 (M)	Heart(17)	TAC MMF	HPN	Fever	Discontinued during the first 11 days and then resumed	moxifloxacin, ganciclovir, IVIG, methylprednisolone, arbidol		N/N	33	Discharged	27
77 (M)	Heart(17)	SIR MMF	HPN DM CVD CKD	Cough, dyspnea	TAC	HCQ, piperacillin, tazobactam, cotrimoxzole, gancyclovir	ARDS	Y/N	12	Discharged	28
67 (M)	Heart(10)	Pred Cysp MMF	HPN	Cough, fever	Pred (R.D.) MMF	HCQ, lopinavir, ritonavir		*/N	23	Discharged	29
38 (M)	Heart(8.7)	TAC MMF Pred		Cough, fever	Pred	HCQ		*/N	21	Discharged	29
43 (M)	Heart(3)	TAC MMF	HPL	Fever	Not reported	ceftriaxone, ganciclovir, moxifloxacin, arbidol		N/N	7	Discharged	27
74 (M)	Heart(23)	TAC, MAM	Cancer UC CVA OSA	Cough, fever	Not reported			N/N	NA	Outpatient	30
39 (M)	Heart and Kidney(3)	TAC MMF Pred	HPN DM	Cough, fever, dyspnea	TAC Pred	HCQ		Y/N	10	Discharged	31

72 (M)	Liver(5.5)	MMF EVE	HPN DM	Cough, fever, dyspnea	TAC	HCQ, lopinavir, ritonavir, interferon-beta	ARDS	*/N	7	Death	26
73 (F)	Liver (16.4)	MMF	DM COPD	Cough, fever, dyspnea			ARDS	Y/Y	24	Death	26
59 (M)	Liver (3)	TAC MMF	Hep B	Cough, fever, dyspnea	TAC MMF (R.D.)	interferon-alpha, lopinavir, ritonavir, piperacillin, tazobactam, methylprednisone, cefperazone-sulbactam, caspofungin, meropenem, coriconazole, IVIG	PNA, cholestasis	Y/Y	45	Death	26
63 (M)	Liver (7.9)	EVE	HPN DM	Cough, fever, dyspnea	TAC MMF			*/N	15	Discharged	26
79 (M)	Liver (15.3)	Pred EVE AZA	DM CKD	Cough, dyspnea	Pred EVE AZA	HCQ, interferon-beta		*/N	14	Discharged	26
63 (M)	Liver (10)	TAC	HPN DM CVD CKD Cancer Hep C	Cough, fever, dyspnea	TAC	HCQ, ceftriaxone, azithromycin, vancomycin, cefepime, tocilizumab		N/N	16	Discharged	32
50 (M)	Liver (3)	TAC	Hep B	Fever		methylprednisone, interferon-alpha, cefoperazone, IVIG, umifenovir, lopinavir, ritonavir		N/N	30	Discharged	33
67 (M)	Liver(19)	Cysp			Cysp			N/N	6	Discharged	30
64 (F)	Liver(13.8)	Pred Cysp MMF	IBD PSC	Cough, fever, dyspnea	Pred (R.D.) MMF	HCQ	ARDS	Y/Y	21	Remain in Hospital	29
76 (M)	Liver(26.5)	TAC	HPN	Fever	TAC	HCQ		N/N	NA	Outpatient	29
46 (F)	Liver(6.4)	TAC			TAC			N/N	NA	Outpatient	29

53 (F)	Lung(20)	Cysp AZA Pred	CVD CKD	Cough, dyspnea	Cysp AZA Pred			N/N	NA	Outpatient	30
59 (F)	Lung (1)	TAC Pred		Cough, dyspnea	No changes	trimethoprim, sulfamethoxazole, metamizole, salbutamol, merpenem		N/N	21	Discharged	34

*ICU care was not explicitly mentioned. **Abbreviations.** ARDS: acute respiratory distress syndrome; AZA: azathioprine; CKD: chronic kidney disease; COPD: chronic obstructive pulmonary disease; CVA: cerebrovascular accident; CVD: cardiovascular disease; Cysp: cyclosporine; EVE: everolimus; HCQ: hydroxychloroquine; HPL: hyperlipidemia; HPN: hypertension; IBD: inflammatory bowel disease; ICU: Intensive care unit; MMF: mycophenolate mofetil; ISx: immunosuppressant; OP: outpatient; OSA: obstructive sleep apnea; Pred: prednisone; PNA: pulmonary nodular amyloidosis; PSC: primary sclerotic cholangitis; R.D.: reduced dose; SIR: Sirolimus; TAC: tacrolimus; UC: ulcerative colitis; Vent: ventilation.

**Table 3:** Summary of characteristics and clinical outcomes of kidney transplant recipients with COVID-19 who continued to use immunosuppressants during hospitalization

	Patients by Age Groups, n (%)					
Clinical Information	All Patients	≤39 Years	40-49 Years	50-59 Years	60-69 Years	≥70 Years
Total No. of patients	89 (100%)	20 (100%)	12 (100%)	24 (100%)	16 (100%)	17 (100%)
No. of patients with a positive outcome	42 (47.2%)	17 (85%)	5 (41.7%)	11 (45.8%)	4 (25%)	5 (29.4%)
No. of patients who remain in hospitals	32 (35.9%)	3 (15%)	7 (58.3%)	8 (33.3%)	10 (62.5%)	4 (23.5%)
No. of patients who died	15 (16.9%)	0	0	5 (20.8%)	2 (12.5%)	8 (47.1%)
No. of patients without comorbidity	24 (100%)	12 (100%)	3 (100%)	2 (100%)	3 (100%)	4 (100%)
No. of patients without comorbidity and who died	2 (8%)	0	0	0	0	2 (40%)
No. of patients with at least 1 comorbidity	65 (100%)	10 (100%)	8 (100%)	22 (100%)	13 (100%)	12 (100)
No. of patients with comorbidity and who died	13 (20%)	0	0	5 (22.7%)	2 (15.4%)	6 (50%)
No. of patients without secondary complication	77 (100%)	17 (100%)	11 (100%)	22 (100%)	12 (100%)	12 (100%)
No. of patient without secondary complication and who died	12 (15.6%)	0	0	4 (18.2%)	2 (16.7%)	6 (35.3%)
No. of patients with a secondary complication	12 (13.5%)	3 (15%)	1 (8.3%)	1 (4.2%)	4 (25%)	3 (17.6%)
No. of patients with a secondary complication and who died	3 (15.6%)	0	0	1 (4.2%)	0	2 (66.7%)
No. of patients without ICU care and/or ventilation	74 (100%)	21 (100%)	10 (100%)	17 (100%)	12 (100%)	14 (100%)
No of patients without ICU care and/or ventilation and who died	10 (13.5%)	0	0	2 (11.8%)	2 (16.7%)	6 (42.8%)
No. of patients with ICU care and/or ventilation	15 (100%)	1 (100%)	2 (100%	7 (100%)	2 (100%)	3 (100%)
No. of patients with ICU care and/or ventilation and who died	5 (33.3%)	0	0	3 (42.9%)	0	2 (66.7%)
No. of female patients	22(100%)	7 (100%)	1 (100%)	4 (100%)	4 (100%)	6 (100%)
No. of female patients who died	5 (21.7%)	0	0	1 (25%)	1 (25%)	3 (50%)
No. of male patients	67 (100%)	13 (100%)	11 (100%)	20 (100%)	12 (100%)	11 (100%)
No. of male patients who died	10 (15.2%)	0	0	4 (20%)	1 (8.3%)	5 (45.5%)

**Table 4:** Ages, comorbidity, and clinical outcomes of kidney transplant recipients with COVID-19 who continued to use immunosuppressants during hospitalization

Patient age (year)	Comorbidity	No. of patients	No. with Positive Outcome	No. Remained Hospitalized	No. Death
≤ 39	No Comorbidity reported	10	9	1	0
	HTN	3	2	1	0
	HTN + DM	3	3	0	0
	HTN + PKD	1	0	1	0
	HTN + Pericarditis	1	1	0	0
	Senior-Loken Syndrome	1	1	0	0
	TTMA	1	1	0	0
40-49	No comorbidity Reported	4	1	3	0
	HTN	5	2	3	0
	HTN + DM	2	1	1	0
	CGN	1	1	0	0
50-59	No Comorbidity reported	3	2	1	0
	HTN	7	1	5	1
	HTN + DM	3	1	0	2
	HCV	1	0	0	1
	HTN + HHD + COPD	1	0	0	1
	HTN + DM + CAD	2	2	0	0
	HTN + CAD	1	1	0	0
	HTN + DM + IHD	1	0	1	0
	HTN + HCV	1	1	0	0
	HTN + Hemolytic anemia	1	1	0	0
	CHD + AF + CHF	1	1	0	0
	PTDM + CMV	1	0	1	0
	RVT + CAD + Testicular cancer	1	1	0	0
60-69	No comorbidity reported	3	1	2	0
	HTN	5	1	3	1
	HTN + DM	4	1	2	1
	HTN + DM + CIN	1	0	1	0
	HTN + DM + IHD	1	0	1	0
	Breast Cancer	1	0	1	0
	CKD3A + MZL + PE + Parkinson + NB	1	1	0	0
≥70	No Comorbidities Reported	5	2	1	2
	HTN	2	1	0	1
	HTN + DM	1	0	1	0
	HTN + DM + CAD	1	1	0	0
	HTN + NRP + DM + PD	1	1	0	0
	HTN + IgA Nephropathy	1	1	0	0
	HTN + DM + NRP	1	0	1	0
	HTN + HD + COPD + Obesity	1	0	0	1
	HTN + Cancer	1	0	0	1
	HTN + PKD + PAC	1	0	0	1
	HTN + ESRD	1	0	0	1
	IHD	1	0	0	1

**Abbreviations.** AF: atrial fibrillation; CAD: chronic allograft dysfunction; CKD3A: chronic kidney disease stage 3A; CGN: chronic glomerulus nephropathy; CHD: chronic heart disease; CHF: congestive heart failure; CMV: cytomegalovirus; COPD: chronic obstructive pulmonary disease; DM: diabetes; ESRD: end stage renal disease; HD: heart disease; HHD: Hypertensive heart disease; HTN: hyertension; IHD: ischemic heart disease; MZL: marginal zone lymphoma; NB: neurogenic bladder; NRP: nephropathy; PAC: prostate adenocarcinoma; PAD: peripheral artery disease; PE: pulmonary embolism; PKD: polycystic kidney disease; PTDM: post-transplantation diabetes mellitus; RVT: renal vein thrombosis; TTMA: transient thrombotic microangiopathy.

**Table 5:** Comorbidities of the kidney transplant recipients with COVID-19 who died

Patient Age and Gender	Comorbidity	In-Hospital Complications	ICU Care/Ventilation	Ref.
50 (M)	HTN, HHD, COPD		/Y	18
56 (M)	HTN, DM		Y/Y	17
56 (F)	HTN, DM	UTI (*E. Coli*)	Y/Y	17
57 (M)	HCV		/	23
59 (M)	HTN		/	23
63 (M)	HTN		/	23
67 (F)	HTN, DM		/	22
70 (F)	HTN		/	23
70 (M)		AKI	/	4
71 (F)	HTN, ESRD		/	21
71 (M)	IHD		/	23
74 (F)	HTN, Cancer		Y/Y	17
75 (M)	HTN, HD, COPD, Obesity		/	11
78 (M)		AKI	/Y	4
78 (M)	HTN, PAC, PKD		/	21

**Abbreviations.** AKI: acute kidney injury; ARDS: acute respiratory distress syndrome; COPD: chronic obstructive pulmonary disease; DM: diabetes; ESRD: end stage renal disease; IHD: ischemic heart disease; HCV: hepatitis C; HD: heart disease; ICU: intensive care unit; PAC: prostate adenocarcinoma; PAD: peripheral artery disease; UTI: urinary tract infection.
