# Supplementary material for: Clinical Outcomes of COVID-19 Patients with Pre-existing, Compromised Immune Systems: A Review of Case Reports
Source: Int J Med Sci. 2020 Oct 18;17(18):2974–86. doi: 10.7150/ijms.50537 (PMC7646123; doi:10.7150/ijms.50537)
Supplement: Supplementary file 1 — Supplementary table. [file ijmsv17p2974s1.pdf]

Supplementary Table 1. Characterization and clinical outcomes of kidney transplant recipients with COVOID-19

Patient Age and Gender	Years Since Transplant	ISx History	Comorbidity	Symptoms on Admission	ISx Used in Hospital	Other Medicines Used in Hospital	In-Hospital Complications	ICU/Vent	Length of Hospital Stay (days)	Clinical Outcome	Ref.
50 (M)	4	TAC<br>MMF<br>Steroids	HTN<br>DM	Fever,<br>Cough	TAC<br>MMF<br>Steroids	Ceftriaxone		/	9	Discharged	35
38 (M)	3	TAC<br>MMF<br>Steroids		Fever,<br>Cough	TAC (R.D.)<br>Steroids	Oseltamavir<br>Arbedol		N/N	16	Discharged	36
38 (M)	5	TAC<br>MMF<br>Steroids	HTN<br>DM	Fever,<br>Cough	TAC<br>MMF<br>Steroids	Oseltamavir<br>Arbedol		N/N	15	Discharged	36
64 (M)	19	TAC<br>MMF<br>Pred		Fever,<br>Cough	TAC<br>Pred			/	N/A	Discharged	37
28 (M)	4	TAC<br>AZA<br>Pred<br>ADM		Fever,<br>Cough	TAC<br>ADM<br>Pred		AKI	/	N/A	Discharged	37
51 (M)	10	TAC<br>MMF<br>Pred		Fever,<br>Cough	TAC<br>Pred		AKI	/	N/A	Discharged	37
32 (F)	1	TAC<br>MMF<br>Pred		Fever,<br>Dyspnea	TAC<br>Pred	HCQ		/	N/A	Discharged	37
21 (M)	4	TAC<br>MMF		Fever,<br>Cough	TAC<br>MMF			/	N/A	Discharged	37
36 (M)	3	BET<br>TAC<br>MMF<br>Pred		Fever,<br>Cough	Betacept<br>TAC<br>Pred	HCQ		/	N/A	Discharged	37
72 (F)	4	TAC		Fever				/	N/A	Discharged	37

		MMF		Cough,<br>Dyspnea							
76 (M)	11	TAC<br>LEF		Fever,<br>Cough	TAC	HCQ		/	N/A	Discharged	37
54 (M)	20	TAC<br>MMF	HTN<br>DM	Fever,<br>Cough	TAC (R.D.)<br>Pred	HCQ<br>AZ<br>Ceftriaxone<br>Chloroquine		/N	13	Discharged	38
61 (M)	15	Cysp<br>Steroid	CKD3A<br>MZL<br>PE<br>Parkinson<br>NB	Fever	Steroid	HCQ<br>AZ<br>Tocilizumab<br>IVIG<br>Meropenem	Pseudomonas	/N	22	Discharged	39
29 (M)	4	Cysp<br>MMF<br>Pred		Fever	Cysp<br>MMF<br>Pred	Lopinavir<br>Ritonavir<br>Moxifloxacin<br>Bactrim<br>Gamma Globulin		/	12	Discharged	40
32 (M)	5	TAC<br>MMF<br>Pred	HTN<br>Pericarditis	Fever,<br>Cough,<br>Dyspnea	TAC<br>MMF<br>Pred	HCQ<br>Oseltamavir<br>Ceftaroline		/	11	Discharged	41
52 (M)	14	TAC<br>MMF<br>Pred		Fever,<br>Cough		IVIG<br>Biapenem<br>Pantoprazole		/	14	Discharged	42
36 (F)	25	TAC<br>Pred	SLS	Cough	Pred	Lopinavir<br>Ritonavir<br>Darunavir<br>Cobicistat<br>Ceftriaxone		/		Discharged	43
49 (M)	9	TAC<br>MMF<br>Pred	CGN	Fever,<br>Cough,<br>Dyspnea	Pred	Ribavirin<br>Umifenovir<br>Moxifloxacin		Y/N	18	Discharged	44
58 (M)	3	BET<br>MMF	TC<br>RVT	Fever,<br>Cough	Pred<br>Cysp			/	18	Discharged	45

		Pred	CAD	Dyspnea							
50 (M)	0	TAC MMF Pred EVE	HTN DM CAD	Cough	TAC MMF EVE			/	N/A	Discharged	46
37 (M)	7	TAC MMF Pred	HTN DM	Fever, Cough	TAC Pred	HCQ AZ		/	N/A	Discharged	46
63 (F)	9	TAC MMF	HTN	Fever, Cough	TAC	HCQ AZ Ceftriaxone	*Enterococcus* Urine	/N	N/A	Discharged	46
30 (F)	4	TAC MMF Pred	HTN DM	Cough	TAC Pred	HCQ AZ Ceftriaxone	*Klebsiella* urine Pneumoniae	/N	N/A	Discharged	46
80 (M)	14	TAC MMF	HTN DM CAD	Fever, Cough		Ceftriaxone Vancomycin		/Y	N/A	Discharged	46
45 (M)	3	TAC MMF Pred	HTN DM	Fever, Cough	TAC Pred	HCQ AZ Ceftriaxone		/N	N/A	Discharged	46
68 (M)	17	TAC MMF Pred	HTN DM	Cough, Dyspnea	TAC Pred	HCQ AZ Ceftriaxone Vancomycin Piperacillin/tac		/N	N/A	Discharged	46
24 (M)		TAC MMF Pred		Fever, Cough	TAC MMF Pred	Antivirals		/	16	Discharged	47
55 (M)		TAC MMF Pred	AF CHD CHF	Cough, Dyspnea	TAC (R.D.) Pred	Antivirals IVIG		/Y	43	Discharged	47
29 (M)		TAC MMF Pred		Fever, Cough, Dyspnea	TAC Pred			/	30	Discharged	47

30 (M)		TAC<br>MMF<br>Pred	HTN	Fever,<br>Cough,<br>Dyspnea	Pred	IVIG		/	16	Discharged	47
50 (M)		TAC<br>MMF<br>Pred	HTN	Fever,<br>Cough,<br>Dyspnea	TAC (R.D.)<br>Pred	Antivirals<br>IVIG		/	26	Discharged	47
52 (M)		TAC<br>MMF<br>Pred	HTN<br>CAD	Fever,<br>Cough,<br>Dyspnea	TAC (R.D.)<br>Pred	Antiviral<br>IVIG		/	13	Discharged	47
49 (M)		TAC<br>MMF		Fever,<br>Cough,<br>Dyspnea	TAC (R.D.)<br>Pred	Antiviral		/	25	Discharged	47
37 (F)		TAC<br>MMF<br>Pred	HTN	Fever,<br>Cough,<br>Dyspnea	TAC (R.D.)<br>Pred	Antiviral<br>IVIG		/	21	Discharged	47
35 (M)	6	EVE<br>Pred		Fever,<br>Cough	Pred<br>Cysp	Chloroquine<br>Liponavir/Ritona<br>vir<br>Loop diuretics<br>Cefuroxime<br>Ceftriaxone		Y/Y	15	Discharged	48
28 (F)	0	TAC<br>MMF<br>Pred	TTMC	Fever	TAC<br>MMF<br>Pred	Oseltamavir<br>AZ		N/N	1	Discharged	49
80 (M)	4	TAC<br>MMF<br>Pred	HTN<br>DM<br>NRP<br>PAD	Cough,<br>Dyspnea	TAC (R.D.)<br>Pred	HCQ<br>Liponavir<br>Ritonavir		/	21	Discharged	26
76 (M)	15	Rap<br>MMF<br>Pred	IgA NRP<br>HTN	Fever	Rap<br>Pred	HCQ		/	10	Discharged	26
48 (M)	21	AZA<br>Pred	HTN		AZA<br>Pred			/	N/A	Discharged	50
54 (M)	6	TAC	Hemolytic		TAC			/	N/A	Discharged	50

		MMF	Anemia<br>HTN								
70 (F)	18	CNI<br>mTORi	HTN	Fever	Steroids	HCQ<br>Lopinavir<br>Ritonavir		/		Discharged	51
51 (M)	23	CNI<br>MMF	HTN<br>HCV	Fever	Steroids	HCQ<br>Lopinavir<br>Ritonavir<br>Tocilizumab		/		Discharged	51
46 (M)	3	CNI<br>MMF	HTN	Fever	Steroids	HCQ<br>Lopinavir<br>Ritonavir		/		Discharged	51
22 (M)	3	TAC<br>Pred		Fever,<br>Cough,<br>Dyspnea	TAC (R.D.)<br>Pred (R.D.)	HCQ<br>AZ<br>Tocilizumab	AKI	/Y	N/A	N/A	37
36 (M)	4	TAC<br>MMF<br>Pred		Fever,<br>Cough	Pred			/N	29	N/A	52
56 (M)	9	TAC<br>Pred	Candida<br>Esophagitis<br>CMV<br>Gastritis<br>AAMR	Cough	TAC (R.D.)<br>Pred	HCQ<br>AZ<br>Famciclovir		/N	18	N/A	52
64 (M)	6	TAC<br>MMF<br>Rap	Breast cancer	Fever,<br>Cough	TAC<br>Steroids	Oseltamavir<br>Arbedol		N/N	27	Still in hospital	36
37 (F)	3	TAC<br>MMF<br>Steroids	HTN	Fever,<br>Cough	Steroids	Oseltamavir<br>Arbedol<br>IVIG		N/N	29	Still in hospital	36
47 (M)	3	TAC<br>MMF<br>Steroids		Fever,<br>Cough		Oseltamavir<br>Arbedol		N/N	18	Still in hospital	36

49 (M)	4	Cysp<br>MMF<br>Pred	HTN<br>DM	Fever,<br>Dyspnea	Cysp<br>MMF<br>Pred	Lopinavir<br>Rionavir<br>Ribavirin<br>Interferon		/N	N/A	Still in hospital	52
51 (F)	1	TAC<br>MMF<br>Pred		Fever,<br>Cough	TAC<br>Pred	HCQ		Y/Y	N/A	Still in hospital	37
61 (M)	0	TAC<br>MMF		Fever,<br>Cough	Pred		AKI	Y/Y	N/A	Still in hospital	37
72 (F)	10	TAC<br>MMF		Fever,<br>Cough	TAC	HCQ<br>AZ		/	N/A	Still in hospital	37
25 (F)	7	TAC<br>MMF<br>Pred		Cough	TAC<br>Pred	HCQ<br>AZ		/	N/A	Still in hospital	37
44 (M)	9	TAC<br>MMF<br>Pred		Dyspnea	TAC (R.D.)<br>Pred	Dialysis	AKI	Y/Y	31	Still in hospital	53
52 (F)	1	TAC<br>MMF<br>Pred	HTN	Fever,<br>Dyspnea	Pred	HCQ<br>Antibiotics<br>Darunavir<br>Cobicistat		N/N	14	Still in hospital	54
50 (M)	4	TAC<br>EVE<br>Pred	HTN	Fever,<br>Cough,<br>Dyspnea	Pred	Ceftriaxone/AZ<br>Liponavir<br>Ritonavir<br>Ceftaroline<br>Meropenem<br>Norepinephrine		Y/Y	12	Still in hospital	55
65 (F)		TAC<br>MMF		Fever,<br>Cough,<br>Dyspnea	TAC (R.D.)<br>Pred	Antiviral<br>IVIG		/Y	45	Still in hospital	47
73 (M)	2	TAC<br>MMF<br>Pred	HTN<br>DM<br>NRP	Fever,<br>Cough,<br>Dyspnea	TAC (R.D.)<br>Pred	HCQ<br>Liponavir<br>Ritonavir<br>IVIG		/	19	Still in hospital	26

71 (M)	30	TAC	HTN<br>DM<br>CAD<br>PKD	Fever	TAC (R.D.)	HCQ<br>IVIG	*Klebsiella* Pneumonia	/	N/A	Still in hospital	26
39 (M)	17	TAC<br>EVE<br>Pred	HTN<br>PKD	Fever	Pred	HCQ<br>Tocilizumab	ARDS	/	15	Still in hospital	26
65 (M)	7	TAC<br>MMF<br>Pred	HTN<br>DM<br>CIN	Fever,<br>Cough,<br>Dyspnea	TAC (R.D.)<br>MMF (R.D.)<br>Pred	HCQ<br>Liponavir<br>Ritonavir	ARDS	/	12	Still in hospital	26
54 (F)	1	TAC<br>MMF<br>Pred	PTDM<br>CMV		Pred			Y/Y	N/A	Still in hospital	50
65 (M)	1	TAC<br>MMF<br>Pred	HTN		TAC<br>Pred			/	N/A	Still in hospital	50
69 (F)	0	TAC<br>MMF<br>Pred	HTN<br>DM		TAC<br>Pred			Y/N	N/A	Still in hospital	50
45 (M)	2	TAC<br>AZA	HTN		TAC (R.D.)			/	N/A	Still in hospital	50
47 (F)	9	CNI<br>MMF<br>Pred		Fever	Pred	HCQ<br>Lopinavir<br>Ritonavir<br>Tocilizumab		/		Still in hospital	51
60 (M)	9	CNI<br>MMF<br>Pred	HTN	Fever	Pred	HCQ<br>Lopinavir<br>Ritonavir		/		Still in hospital	51
73 (M)	7	CNI<br>MMF<br>Pred	HTN<br>DM	Fever	Pred	HCQ<br>Lopinavir<br>Ritonavir		/		Still in hospital	51
59 (M)	10	MMF<br>Pred	HTN<br>DM<br>IHD	Fever	Pred	HCQ<br>Lopinavir<br>Ritonavir		/		Still in hospital	51

49 (M)	2	CNI<br>MMF<br>Pred	HTN	Fever	Pred	HCQ<br>Lopinavir<br>Ritonavir<br>Tocilizumab		/		Still in hospital	51
60 (F)	2	CNI<br>MMF<br>Pred	HTN	Fever	Pred	HCQ<br>Lopinavir<br>Ritonavir		/		Still in hospital	51
57 (M)	11	CNI<br>MMF	HTN	Fever	Steroids	HCQ<br>Lopinavir<br>Ritonavir		/		Still in hospital	51
54 (M)	18	CNI<br>Pred	HTN	Fever	Pred	HCQ<br>Ritonavir<br>Darunavir		/		Still in hospital	51
60 (M)	13	CNI	HTN<br>DM<br>IHD	Fever	Steroids	HCQ<br>Lopinavir<br>Ritonavir		/		Still in hospital	51
50 (M)	10	CNI<br>MMF<br>Pred	HTN	Fever	Pred	HCQ<br>Ritonavir<br>Darunavir		/		Still in hospital	51
69 (M)	22	CNI<br>Pred	HTN<br>DM	Fever	Pred	HCQ<br>Ritonavir<br>Darunavir		/		Still in hospital	51
44 (M)	14	CNI<br>mTORi	HTN	Fever	Steroids	Darunavir		/		Still in hospital	51
70 (M)	5	BET<br>MMF<br>Pred		Fever,<br>Cough	Pred		AKI	/	N/A	Death	37
78 (M)	10	TAC<br>MMF<br>Pred		Cough,<br>Dyspnea	TAC<br>Pred	HCQ<br>AZ	AKI	/Y	N/A	Death	37
75 (M)	10	TAC<br>MMF<br>Pred	COPD<br>HD<br>HTN	Fever,<br>Dyspnea	Pred	HCQ<br>Lopinavir<br>Ritonavir		N/N		Death	54

			Obesity			Antibiotics					
56 (M)	21	TAC<br>MMF<br>Pred	HTN<br>DM	Fever,<br>Cough	Pred	HCQ<br>AZ<br>Ceftriaxone		Y/Y	N/A	Death	46
74 (F)	9	SIR<br>Pred	HTN<br>Cancer	Fever,<br>Cough,<br>Dyspnea	Pred	HCQ<br>AZ		Y/Y	N/A	Death	46
56 (F)	3	TAC<br>MMF	HTN<br>DM	Fever,<br>Cough,<br>Dyspnea	TAC	HCQ<br>Levofloxacin	*E. Coli* Urine	Y/Y	N/A	Death	46
50 (M)		Cysp<br>MIZ	HTN<br>HHD<br>COPD	Fever,<br>Cough,<br>Dyspnea	TAC (R.D.)<br>Pred	Antiviral<br>IVIG		/Y	6	Death	47
78 (M)	8	TAC<br>Pred	PKD<br>HTN<br>PAC	Fever,<br>Dyspnea	TAC (R.D.)<br>Pred	Liponavir<br>Ritonavir		/	4	Death	26
71 (F)	6	TAC<br>MMF<br>Pred	ESRD<br>HTN	Fever,<br>Cough,<br>Dyspnea	TAC (R.D.)<br>Pred	HCQ<br>Liponavir<br>Ritonavir<br>IVIG		/	9	Death	26
67 (F)	1	TAC<br>MMF<br>Pred	HTN<br>DM		TAC<br>Pred			/	12	Death	50
71 (M)	13	CNI<br>MMF<br>Pred	IHD	Fever	Pred	HCQ<br>Lopinavir<br>Ritonavir		/		Death	51
57 (M)	2	CNI<br>MMF<br>Pred	HCV	Fever	Pred	HCQ<br>Lopinavir<br>Ritonavir<br>Tocilizumab		/		Death	51
59 (M)	5	CNI<br>MMF<br>Pred	HTN	Fever	Pred	HCQ<br>Lopinavir<br>Ritonavir		/		Death	51
70 (F)	16	CNI	HTN	Fever	Pred	HCQ		/		Death	51

		Pred				Lopinavir<br>Ritonavir					
63 (M)	16	MMF<br>Pred	HTN	Fever	Pred	HCQ<br>Lopinavir<br>Ritonavir<br>Tocilizumab		/		Death	51

Abbreviations. AAMR: acute antibody mediated rejection; AF: atrial fibrillation; AKI: acute kidney injury; ARDS: acute respiratory distress syndrome; AZ: azithromycin; CAD: chronic allograft dysfunction; BET: betacept; CGN: chronic glomerulus nephropathy; CHF: congestive heart failure; CHD: chronic heart disease; CKD3A: chronic kidney disease stage 3A; CMV: cytomegalovirus; ESRD: end stage renal disease; HD: heart disease; CKD: chronic kidney disease; CIN: chronic interstitial nephritis; CNI: calcinuerin inhibitor; COPD: chronic obstructive pulmonary disease; CVA: cerebrovascular accident; CVD: cardiovascular disease; Cysp: cyclosporine; EVE: everolimus; HCQ: hydroxychloroquine; HCV: hepatitis C; HPL: hyperlipidemia; HPN: hypertension; IBD: inflammatory bowel disease; ICU: Intensive care unit; IHD: ischemic heart disease; ISx: immunosuppressant; LEF: Leflunomide; MIZ: mizoribine; MMF: mycophenolate mofetil; MZL: marginal zone lymphoma; mTORi: mTOR inhibitor; NB: neurogenic bladder; NRP: nephropathy; OP: outpatient; OSA: obstructive sleep apnea; PAC: prostate adenocarcinoma; PAD: peripheral artery disease; PE: pulmonary embolism; PKD: polycystic kidney disease; Pred: prednisone; PNA: pulmonary nodular amyloidosis; PSC: primary sclerotic cholangitis; PTDM: post-transplantation diabetes mellitus R.D.: reduced dose; RVT: renal vein thrombosis; SIR: Sirolimus; SLS: Senior-Loken syndrome; TAC: tacrolimus; TC: testicular cancer; TTMA: transient thrombotic microangiopathy; UC: ulcerative colitis; Vent: ventilation.
